# Baicalein 5,6-Dimethyl Ether Prevents Memory Deficits in the Scopolamine Zebrafish Model by Regulating Cholinergic and Antioxidant Systems

**DOI:** 10.3390/plants10061245

**Published:** 2021-06-18

**Authors:** Ion Brinza, Iriny M. Ayoub, Omayma A. Eldahshan, Lucian Hritcu

**Affiliations:** 1 Department of Biology, Faculty of Biology, Alexandru Ioan Cuza University of Iasi, 700506 Iasi, Romania; ion.brinza@student.uaic.ro; 2 Department of Pharmacognosy, Faculty of Pharmacy, Ain Shams University, Abbassia, Cairo 11566, Egypt; irinyayoub@pharma.asu.edu.eg; 3 Center of Drug Discovery Research and Development, Ain Shams University, Abbassia, Cairo 11566, Egypt

**Keywords:** *Alnus rugosa*, baicalein 5,6-dimethyl ether, memory, anxiety, oxidative stress, scopolamine, zebrafish

## Abstract

Baicalein 5,6-dimethyl ether, a bioactive flavonoid isolated for the first time from *Alnus rugosa*, was explored for its capability to relieve memory deficits and decrease oxidative stress. We examined the neuropharmacological effects of baicalein 5,6-dimethyl ether on scopolamine (Sco)-induced zebrafish (*Danio rerio*) anxiety, amnesia, and brain oxidative stress and attempted to elucidate the underlying mechanisms. Anxiety-like behavior, exploratory behavior, and memory performance were measured using novel tank-diving test (NTT), Y-maze, and novel object recognition (NOR) tests. For 10 days, baicalein 5,6-dimethyl ether (1, 3, and 5 µg/L) was administered through immersion, whereas Sco (100 μM) was delivered 30 min before behavioral tests. Treatment with baicalein 5,6-dimethyl ether reduced anxiety and memory impairment, and increased exploratory behavior in specific tests, along with significant protection from neuronal oxidative stress in the brain tissue of Sco-treated zebrafish. Antioxidant and anti-acetylcholinesterase (AChE) activities of baicalein 5,6-dimethyl ether in the Sco-induced zebrafish were further confirmed using in vivo assays. In Sco-treated zebrafish, baicalein 5,6-dimethyl ether regulated cholinergic function by inhibiting AChE activity. Baicalein 5,6-dimethyl ether may be a promising candidate compound for treating anxiety and amnesia by restoring cholinergic activity and reducing brain oxidative stress, according to our findings.

## 1. Introduction

*Alnus* is a genus belonging to the family Betulaceae widely distributed in Africa, Europe, Asia, and North America. *Alnus* comprises more than 40 species [1]. *Alnus* species are recognized in folk medicine for the treatment of a variety of diseases including cancer, inflammation, hepatitis, rheumatism, stomachache, diarrhea, dysentery, fever, etc. [2].

Diarylheptanoids, along with polyphenols, flavonoids, steroids, and terpenoids have been reported in genus *Alnus* [2]. *Alnus rugosa* (Du Roi) Spreng. (Betulaceae) is used in traditional medicine as an astringent, cathartic, emetic, tonic, anodyne, and febrifuge [3]. Aerial parts of *A. rugosa* showed antimicrobial and cytotoxic activities [4]. *A. rugosa* stem methanolic extract was reported to possess anticholinesterase activity [5].

Baicalein was originally isolated from the roots of *Scutellaria baicalensis* Georgi, the traditional Chinese herb Huangqin. It has been widely used in China and South Korea to treat cancer and inflammatory diseases [6]. Baicalein exhibits a myriad of biological activities, including antioxidant, free radical scavenging activities, xanthine oxidase inhibition, as well as 12/15-lipoxygenase inhibition [7]. Moreover, baicalein possesses antidiabetic and anti-inflammatory properties [8]. It was reported to antagonize the adhesion molecule expression induced by interleukin-β1 and tumor necrosis factor (TNF-α) [9]. Interestingly, baicalein exhibits potent neuroprotective activity [10]. Baicalein showed neuroprotective effects in 6-hydroxydopamine-induced parkinsonism and proved to be a promising candidate for the prevention or treatment of Parkinson’s disease, owing to its anti-inflammatory, anti-apoptotic, and pro-differentiation action [11]. Furthermore, baicalein exhibited a neuroprotective activity against amnesia induced by β-amyloid peptide-(25-35) (Aβ25-35) [12] and showed therapeutic potential for the treatment of Alzheimer’s disease (AD) [13]. Moreover, it served as a neuroprotective against neuronal injury secondary to ischemic insult [14]. Baicalein protected the rat brain against ischaemic/reperfusion injury via downregulation of the lectin-like oxidized low-density lipoprotein receptor-1 (LOX-1) and nuclear factor-kB (NF-kB) expression and the AMP-activated protein kinase/nuclear factor erythroid 2-related factor 2 (AMPK/Nrf2) pathway [15]. Recently, baicalein was reported to exert an inhibitory effect on ferroptosis in FeCl_3_-induced post-traumatic epileptic seizures [16]. Baicalein showed promising antiviral activities including anti-human immunodeficiency virus (HIV) [17], anti-severe acute respiratory syndrome (SARS) coronavirus [18], anti-dengue virus (DENV) [19], anti-chikungunya virus (CHIKV) [20] and anti-influenza virus [21], anti-Zika virus [22], and anti-Herpes simplex virus type 1 (HSV-1) [23].

Scopolamine (Sco) is used in animal models to simulate cognitive inadequacy [24]. Because of their high genomic similarities to humans [25] and the involvement of classical neurotransmitter systems linked to memory and learning processing, zebrafish are now used as an animal model for researching complex behaviors in the modern world [26,27]. Sco causes cognitive impairment and disrupts memory processing in zebrafish, according to several published reports [28,29,30].

In the background of this information, in the present study, we indicated that mechanisms of neuroprotection and antioxidation of baicalein 5,6-dimethyl ether, a derivative of baicalein isolated from *A. rugosa* (Betulaceae), in Sco-injected zebrafish, are regulated by the cholinergic system activation. Therefore, the current study proposes that baicalein 5,6-dimethyl ether could be an encouraging agent and serve as a potent neuropharmacological drug candidate against the dementia-related condition.

## 2. Results

### 2.1. Isolation and Structure Elucidation of the Isolated Compound

Compound 1 was obtained as a yellow, amorphous powder. The electrospray-ionization mass spectrometry (ESIMS) data of compound 1 showed a protonated molecular ion at m/z 299 [M + H]^+^ in the positive ion mode. The UV spectrum of compound 1 exhibited absorption maxima at 244, 263, and 320 nm, suggesting a flavone [31,32]. The ^1^H NMR spectrum of compound 1 suggested a flavone skeleton. Three multiplets integrating for five protons at δH 8.07, 8.01, and 7.56 designated the presence of an unsubstituted B-ring [33]. Two aryl methoxy groups were identified herein, exhibiting ^13^C NMR chemical shifts at 59.54, 59.45 ppm. The ^13^C NMR chemical shifts of arylmethoxy groups were indicative of their conformation, where a coplanar methoxy group usually exhibits a signal between 55 and 57 ppm; however, a methoxy group adopts an out-of-plane conformation when located between two adjacent substituents, exhibiting a characteristic 5–7 ppm downfield chemical shift [34], thus, suggesting a 5, 6-dimethoxy derivative. Therefore, compound 1 was identified as baicalein 5,6-dimethyl ether, which was confirmed by APT, HSQC, HMBC, and COSY experiments. This is the first report of baicalein 5–6-dimethyl ether in genus *Alnus*.

### 2.2. The Effect of Baicalein 5,6-Dimethyl Ether on Scopolamine-Induced Anxiety in Zebrafish

There was no toxicity in any study group in terms of general behavioral changes or mortality, and no adverse effects were found.

**Figure 1:**
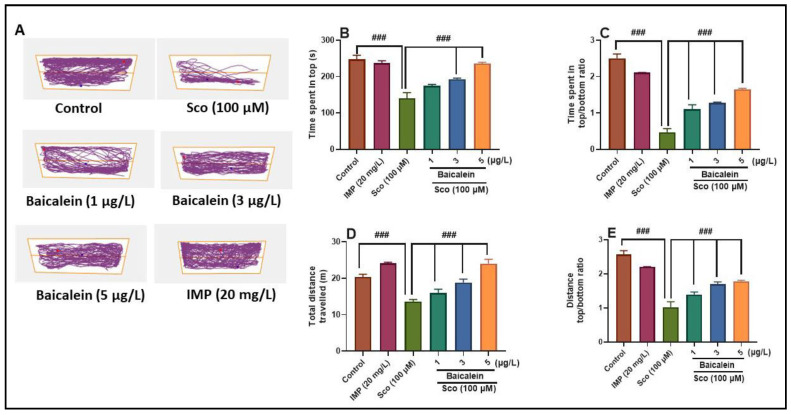
Effects of the baicalein 5,6-dimethyl ether (1, 3, and 5 µg/L) on scopolamine (Sco, 100 μM)-induced anxiety-like behavior in the novel tank-diving test (NTT). (**A**) representative swimming tracks of zebrafish in each group; (**B**) the time spent in top (s) in each group; (**C**) time spent in top/bottom ratio in each group; (**D**) total distance travelled (m) in each group; (**E**) distance top/bottom ratio in each group. Values are means ± S.E.M. (*n* = 10). For Tukey’s post hoc analyses: Control vs. Sco: ### *p* < 0.0001, Sco vs. Baicalein 5,6-dimethyl ether (1, 3, and 5 µg/L): ### *p* < 0.0001 (**B**–**E**).

The NTT test was performed to determine whether baicalein 5,6-dimethyl ether treatment attenuates anxiety-like response induced by Sco. Representative swimming tracks of zebrafish (Figure 1A) in Sco-induced zebrafish indicated a high level of anxiety, as evidenced by increased exploration of the bottom zone of the tank, as compared to the control group. Moreover, intense exploratory behavior following baicalein 5,6-dimethyl ether treatment in the Sco group was noticed. One-way ANOVA revealed significant overall changes of the time spent in top (F (5, 54) = 24.97, *p* < 0.0001) (Figure 1B), the time spent in top/bottom ratio (F (5, 54) = 78.98, *p* < 0.0001) (Figure 1C), total distance travelled (F (5, 54) = 24.85, *p* < 0.0001) (Figure 1D), and distance top/bottom ration (F (5, 54) = 38.02, *p* < 0.0001) (Figure 1E). As shown in Figure 1, Sco (100 µM)-treated zebrafish showed a higher anxiogenic profile, as evidenced by a significant decrease of the time spent in tope zone of the tank (*p* < 0.0001) (Figure 1B), the time spent in top/bottom ratio (*p* < 0.0001) (Figure 1C), total distance travelled (*p* < 0.0001) (Figure 1D), and distance top/bottom ration (*p* < 0.0001) (Figure 1E), when compared to the control group. Sco-induced zebrafish treated with IMP (20 mg/L), a well-known tricyclic antidepressant, used a positive control, showing better performance than the zebrafish injected with Sco alone in the NTT. Furthermore, baicalein 5,6-dimethyl ether dose-dependently reduced the effects of Sco (*p* < 0.0001) as compared to Sco-alone treated zebrafish, implying that it has anxiolytic properties.

Our data agree with the literature where baicalein administration attenuated depressive- and anxiety-like behavior. Baicalein attenuated depressive-like behavior in mice by inhibiting neuroinflammation via downregulation of the NF-κB pathway as reported by Du et al. [35]. De Carvalho et al. [36] reported that baicalein promoted the anxiolytic-like and sedative effects in mice, pharmacological activities dependent on GABAergic non-benzodiazepine sites but not on the serotonin (5-HT) system. Wang et al. [12] reported that baicalein may act on the benzodiazepine binding sites to exert an anxiolytic-like effect in mice. Liao et al. [37] concluded that the anxiolytic-like effect of baicalein or baicalin may be mediated through activation of the benzodiazepine binding site of GABA(A) receptors. Additionally, some evidence suggested that Sco induced conflicting results. Hamilton et al. [38] reported the anxiolityc effects of Sco (800 µM) in zebrafish, as evidenced by their decreased tendency to shoal. Besides, not all zebrafish research supports Sco’s anxiolityc profile. In an earlier investigation in adult zebrafish [39], Sco was found to have no anxiolityc effects while also suppressing the anxiolotyc-like effect of physostigmine, an AChE inhibitor. Oppositely to Hamilton et al. [38], but in line with the rodent studies suggesting the anxiogenic effects of Sco via disruption of the cholinergic functions [40,41], the present study reported the anxiogenic profile of the Sco, leading to increased preference to explore of the bottom zone of the tank in the NTT test.

On these results, our data indicated that baicalein 5,6-dimethyl ether significantly counters the Sco-induced anxiety in the zebrafish model.

### 2.3. The Effect of Baicalein 5,6-Dimethyl Ether on Scopolamine-Induced Deficits of Exploratory Behavior and Recognition Memory in Zebrafish

**Figure 2:**
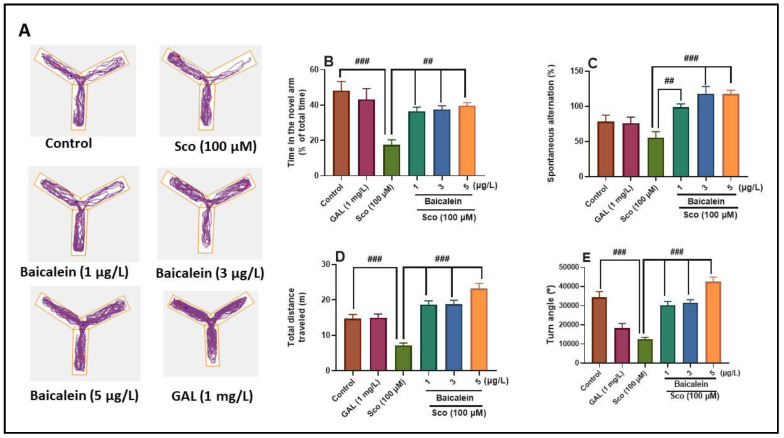
Effects of the baicalein 5,6-dimethyl ether (1, 3, and 5 µg/L) on scopolamine (Sco, 100 μM)-induced increasing of the exploratory behavior and locomotion in the Y-maze. (**A**) representative swimming tracks of zebrafish in each group; (**B**) time in the novel arm (% of the total time) in each group; (**C**) spontaneous alternation (%) in each group; (**D**) total distance travelled (m) in each group; (**E**) turn angle (°) in each group. Values are means ± S.E.M. (*n* = 10). For Tukey’s post hoc analyses: Control vs. Sco: ### *p* < 0.0001, and ## *p* < 0.001, Sco vs. Baicalein 5,6-dimethyl ether (1, 3, and 5 µg/L): ## *p* < 0.001, and ### *p* < 0.0001 (**B**–**E**).

The Y-maze was used to investigate the effects of baicalein 5,6-dimethyl ether on the tendency of animals to explore new environments. Tracking plots of the fish exposed to Sco indicated deficits of the response to explore new environments, as evidenced by reduced activity in the novel arm (Figure 2A). However, the administration of baicalein 5,6-dimethyl ether and GAL significantly increased the activity in the novel arm of the Y-maze. The results of one-way ANOVA revealed significant overall effects of treatment on the time in the novel arm (F (5, 54) = 7.87, *p* < 0.0001) (Figure 2B), the spontaneous alternation percentage (F (5, 54) = 9.71, *p* < 0.0001) (Figure 2C), the total distance travelled (F (5, 54) = 24.34, *p* < 0.0001) (Figure 2D), and the turn angle (F (5, 54) = 25.20, *p* < 0.0001) (Figure 2E). As Figure 2 shows, Sco significantly impaired the response to novelty, as evidenced by decreased time in the novel arm (*p* < 0.0001) (Figure 2B) and the spontaneous alternation percentage (*p* < 0.001) (Figure 2C), as compared to the control group. Moreover, the Sco-injected zebrafish exhibited a hypolocomotory activity, as evidenced by a significant decrease of the total distance travelled (*p* < 0.0001) (Figure 2D) and the turn angle (*p* < 0.0001) (Figure 2E), as compared to the control group. By contrast, GAL and baicalein (1, 3, and 5 µg/L) promoted an exploratory-enhancing effect as noticed by increasing the time in the novel arm (*p* < 0.001) (Figure 2B) and the spontaneous alternation percentage (*p* < 0.001 for 1 µg/L and *p* < 0.0001 for 3 and 5 µg/L) (Figure 2C) in the Sco-injected zebrafish compared to Sco-alone treated groups. Besides, GAL and baicalein (1, 3, and 5 µg/L) increased the locomotion profile of the Sco-treated zebrafish, as evidenced by a significant increase of the total distance travelled (*p* < 0.0001) (Figure 2D), and the turn angle (*p* < 0.0001) (Figure 2E).

**Figure 3:**
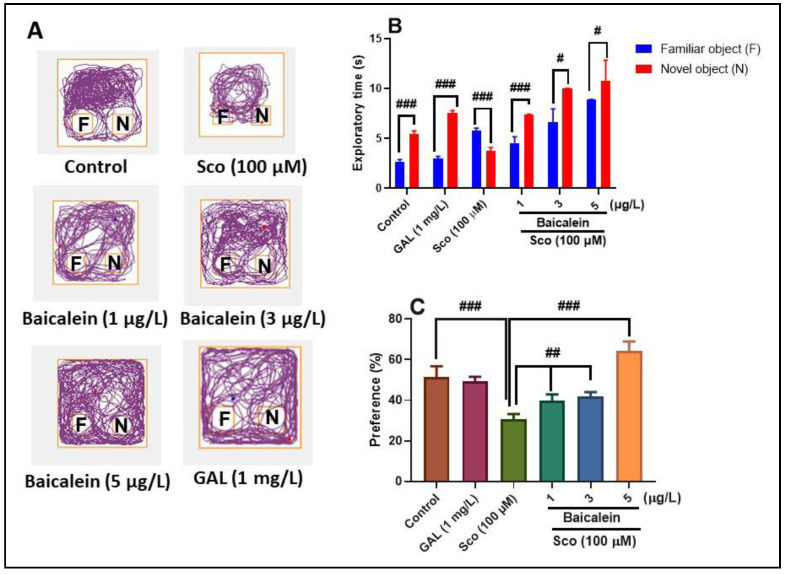
Baicalein 5,6-dimethyl ether (1, 3, and 5 μg/L) improved recognition memory in the novel object recognition (NOR) test. (**A**) Representative locomotion-tracking pattern of the control, scopolamine (Sco: 100 μM), baicalein 5,6-dimethyl ether (1, 3, and 5 μg/L), and galantamine (GAL, 1 mg/L) treated groups; the familiar object—F and the novel object (N); (**B**) Exploratory time of the familiar object (F) and novel object (N) in different groups; (**C**) The percentages of preference of the familiar object (F) and novel object (N) in different groups. Values are means ± S.E.M. (*n* = 10). Tukey’s post hoc analyses: (**B**) ### *p* < 0.0001 and # *p* < 0.01; (**C**) ### *p* < 0.0001 and ## *p* < 0.001.

The novel object recognition test (NOR) exploits the tendency of most vertebrates to explore novel objects over familiar ones [42]. Representative tracking plots of Sco-treated zebrafish indicated deficits in recognition memory, as evidenced by reducing the exploration of the novel object rather than the familiar object. However, the administration of baicalein 5,6-dimethyl ether and GAL significantly increased the preference for the novel object in NOR (Figure 3A). One-way ANOVA showed significant overall effects of the treatment on the exploratory time (F (5, 54) = 23.11, *p* < 0.0001) (Figure 3B) and the percentage of preference (F (5, 54) = 10.86, *p* < 0.0001) (Figure 3C).

The control group showed a significant preference to explore the N (*p* < 0.0001), in contrast with Sco-treated fish that explored F rather than N (*p* < 0.0001), suggesting an impairment of recognition memory (Figure 3B). Administration of baicalein 5,6-dimethyl ether (1, 3, and 5 µg/L) to the Sco-injected fish resulted in increasing preference for N instead of F (*p* < 0.0001 for 1 µg/L and *p* < 0.01 for 3 and 5 µg/L), indicating recognition memory improvement effects (Figure 3B). The percentage of preference to explore N significantly decreased following Sco administration (*p* < 0.0001) (Figure 3C) in zebrafish compared to the control group, whereas the Sco-injected zebrafish subjected to baicalein 5,6-dimethyl ether treatment (1, 3, and 5 µg/L) displayed high percentages of preference to explore N (*p* < 0.001 for 1 and 3 µg/L and *p* < 0.0001 for 5 µg/L), suggesting memory improvement.

Our results indicated that baicalein 5,6-dimethyl ether promoted a cognitive-enhancing profile, which agrees with the literature where baicalein significantly attenuated memory decline. Baicalein is considered a potent compound for the treatment of AD, by preventing cholinergic dysfunction, brain oxidative stress, and cognitive deficits, which is caused by Sco. This outcome may be partially due to the upregulation of DHCR24, SELADIN, and SIRT6 in the entire hippocampal region in the rat model of AD [43]. Li et al. [44] suggested that baicalein could delay senescence and improve cognitive dysfunction in senescence-accelerated mouse prone 8 (SAMP8) employing a mechanism dependent upon the inhibition of Aβ1-42 and RAGE/JAK2/STAT1 cascade. Li et al. [45] investigated the neuroprotective effects of baicalein and the effect of the cortical 12/15-lipoxygenase (12/15-LOX) pathway on diabetic cognitive dysfunction. The authors have shown that baicalein improved the cognitive function of diabetic rats by directly acting in the brain, rather than by regulating the levels of blood glucose, lipids, or insulin. In addition, baicalein protected rat cortical neurons from damage caused by diabetes via inhibiting the 12/15-LOX pathway and relieving inflammation and apoptosis of the central nervous system. Wang and Zhou [46] demonstrated that baicalein reduced neurodegeneration and improved learning and memory retention of rats and as well-modulated PI3/Akt/GSK-3β and JNK/ERK signaling pathways. Also, baicalein improved senescence status and improves cognitive function in SAMP8 mice, and that this effect might be attributable to suppression of cortical proinflammatory cytokines and modulation of the intestinal microbiome, as reported by Gao et al. [47]. Finally, it has been reported that baicalein improved learning and memory dysfunction in d-galactose-induced aging rats. This might be achieved through attenuation of inflammation and metabolic dysfunction [48]. These results provide strong evidence that baicalein 5,6-dimethyl ether, investigated herein, could improve exploratory behavior and memory performance in a Sco zebrafish model, as evidenced in the present study.

### 2.4. In Vivo Inhibitory Activity of Baicalein 5,6-Dimethyl Ether Against Acetylcholinesterase Activity

**Figure 4:**
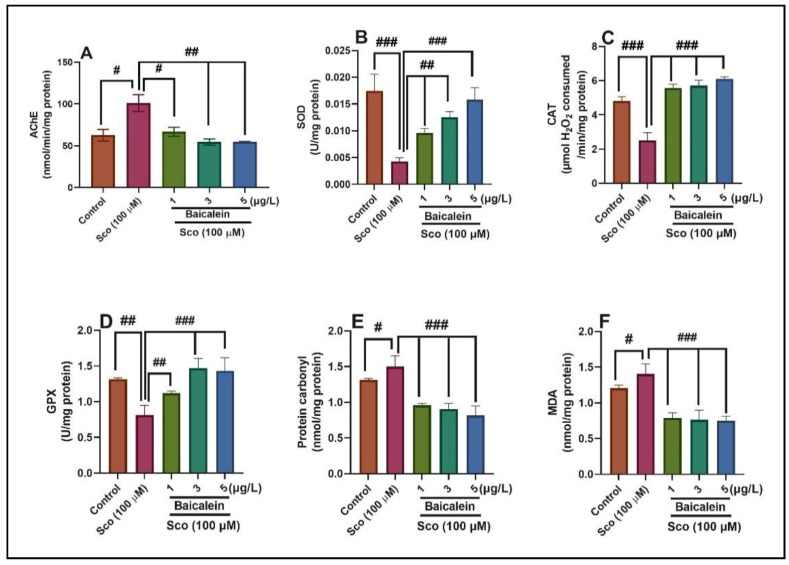
Effects of the baicalein 5,6-dimethyl ether on (**A**) AChE; (**B**) superoxide dismutase (SOD); (**C**) catalase (CAT) and (**D**) glutathione peroxidase (GPX) specific activities; (**E**) protein carbonyl and (**F**) malondialdehyde (MDA) levels. Values represent means ± S.E.M. (*n* = 10) followed by Tukey’s *post hoc* analyses: (**A**) # *p* < 0.01 and ## *p* < 0.001; (**B**) ### *p* < 0.0001 and ## *p* < 0.001; (**C**) ### *p* < 0.0001; (**D**) ## *p* < 0.001 and ### *p* < 0.0001; (**E**) # *p* < 0.01 and ### *p* < 0.0001; (**F**) # *p* < 0.01 and ### *p* < 0.0001.

The brain acetylcholinesterase (AChE) activity was assessed to further elucidate the possible mechanism of baicalein 5,6-dimethyl ether against Sco-induced memory deficits. One-way ANOVA revealed significant overall effects of the treatment on AChE activity (F (4, 45) = 9.38, *p* < 0.01) (Figure 4A). Figure 4A shows a significant increase of the AChE activity (*p* < 0.01) in the Sco-injected fish compared to the control group. However, Sco-treated zebrafish that received baicalein 5,6-dimethyl ether showed a significant dose-depending decrease of AChE activity (*p* < 0.01 for 1 μg/L and *p* < 0.001 for 1 and 5 μg/L) compared to Sco-alone treated fish. These outcomes suggest that baicalein 5,6-dimethyl ether could protect against Sco-induced dysfunction of the cholinergic system.

Supporting data provide evidence of the anti-AChE activity of baicalein. Janjusevic et al. [49] demonstrated that flavonoids baicalein and quercetin identified in the *Trametes versicolor* water extract may be responsible for the observed AChE inhibitory activity. Zhou et al. [50] showed that baicalein improved behavioral dysfunction induced by AD in rats, using decreasing AChE level. On these results, we could suggest that baicalein improved memory processes in Sco-treated fish by restoring the cholinergic function, implying inhibition of AChE activity.

### 2.5. In Vivo Activity of Baicalein 5,6-Dimethyl Ether on the Antioxidant Defense System

The effects of baicalein 5,6-dimethyl ether on antioxidant enzymes (e.g., SOD, CAT, GPX) specific activities and protein oxidation and lipid peroxidation contents in the zebrafish brains were evaluated to further examine the mechanism involved in preventing memory decline. The results of the one-way ANOVA demonstrated overall significant effects of the treatment on SOD (F (4, 45) = 7.99, *p* < 0.0001) (Figure 4B), CAT (F (4, 45) = 24.49, *p* < 0.0001) (Figure 4C), and GPX (F (4, 45) = 4.98, *p* < 0.0001) (Figure 4D) specific activities. Administration of Sco reduced the specific activity of the antioxidant enzymes such as SOD (*p* < 0.0001) (Figure 4B), CAT (*p* < 0.0001) (Figure 4C), and GPX (*p* < 0.001) (Figure 4D), compared to the control groups, whereas treatment with baicalein 5,6-dimethyl ether dose-dependently reversed the reduction of SOD (*p* < 0.001 for 1 and 3 µg/L and *p* < 0.0001 for 5 µg/L) (Figure 4B), CAT (*p* < 0.0001 for 1, 3, and 5 µg/L) (Figure 4C) and GPX (*p* < 0.001 for 1 µg/L and *p* < 0.0001 for 3, 5 µg/L) (Figure 4D). Also, one-way ANOVA revealed significant effects of treatment on protein carbonyl (F (4, 45) = 8.87, *p* < 0.001) (Figure 4E) and MDA (F (4, 45) = 9.15, *p* < 0.001) (Figure 4F) contents. Levels of protein carbonyl (protein oxidation) and MDA (lipid peroxidation) was significantly elevated (*p* < 0.01) in Sco-treated fish compared to the control groups. In contrast, the administration of baicalein 5,6-dimethyl ether prevented the formation of protein carbonyl and MDA in Sco-induced zebrafish.

The literature outcomes supported that baicalein exerted neuroprotective effects by inhibiting oxidative stress. Qian et al. [51] demonstrated that the neuroprotective effects of baicalein on cognition and the hippocampus are associated with the suppression of oxidative stress and inflammation and the regulation of the glucocorticoid pathway and actin-associated protein in temporal lobe epilepsy rats. Moreover, it has been reported that baicalein promoted neuronal and behavioral recovery after intracerebral hemorrhage by suppressing oxidative stress [52]. Therefore, these results suggested that baicalein 5,6-dimethyl ether could protect the brain against Sco-induced oxidative stress by regulating the activity of antioxidant enzymes along with protein oxidation and lipid peroxidation contents.

### 2.6. Correlation between Behavioral Scores, Enzymatic Activities, and Lipid Peroxidation

**Figure 5:**
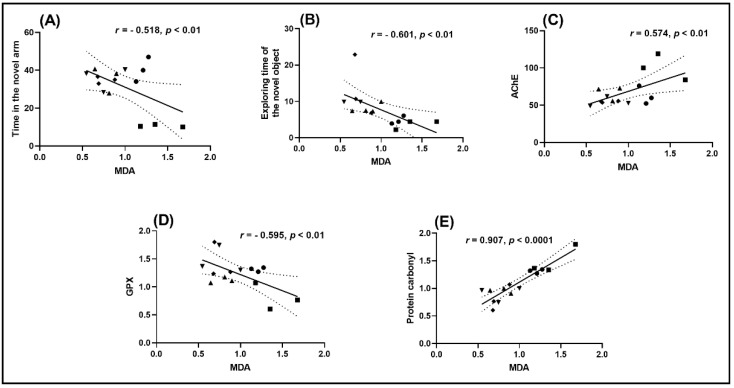
Correlation analyses between behavioral and biochemical parameters (Pearson’s correlation). Data expressed are time in the novel arm (s), exploring the time of the novel object (s), AChE (nmol/min/mg protein), GPX (U/mg protein), protein carbonyl (nmol/mg protein), and MDA (nmol/mg protein). (**A**) Time in the novel arm vs. MDA (*n* = 10, *r* = −0.518, *p* < 0.01); (**B**) Exploring time of the novel object vs. MDA (*n* = 10, *r* = −0.601, *p* < 0.01); (**C**) AChE vs. MDA (*n* = 10, *r* = 0.574, *p* < 0.01); (**D**) GPX vs. MDA (*n* = 10, *r* = −0.595, *p* < 0.01) and (**E**) protein carbonyl vs. MDA (*n* = 10, *r* = 0.907, *p* < 0.0001) in control (●), scopolamine (Sco) (■), and baicalein 5,6-dimethyl ether ((▲) 1, (▼) 3, and (♦) 5 μg/L) groups.

Pearson’s correlation coefficient (*r*) was used to evaluate the relationship between the behavioral scores, enzymatic activities, and lipid peroxidation, including time in the novel arm, exploring time of the novel object, AChE, GPX, protein carbonyl, and MDA (Figure 5). The time in the novel arm (Figure 5A) and the exploring time of the novel object (Figure 5B) showed a significant negative correlation to MDA with *r* of −0.518 (Figure 5A) and −0.601 (Figure 5B), respectively, while a high negative correlation between GPX vs. MDA (Figure 5D) was noticed with *r* of −0.595. However, a positive correlation between AChE vs. MDA (Figure 5C) and protein carbonyl vs. MDA (Figure 5E) was observed with *r* of 0.574 (Figure 5C) and 0.907 (Figure 5E), respectively.

Gao et al. [47] demonstrated a positive correlation between cognitive abilities and suppression of cortical proinflammatory cytokines and modulation of the intestinal microbiome in SAMP6 mice following administration of baicalein. Additionally, Kim et al. [53] indicated a significant decrease of oxidative stress correlated with protective effects against DNA damage. We used the determination of the *r* to show that improved memory output in Sco-treated zebrafish is related to increased antioxidant enzyme activity and lower MDA (lipid peroxidation levels), confirming the baicalein 5,6-dimethyl ether neuroprotective profile.

## 3. Materials and Methods

### 3.1. Plant Material

*A. rugosa* leaves were collected from Al Zohriya Botanical Garden in Egypt, kindly authenticated by Dr. Usama K. Abdel Hameed in the Department of Botany, Faculty of Science, Ain Shams University, Cairo, Egypt. A voucher specimen was deposited in the herbarium of Pharmacognosy Department, Faculty of Pharmacy, Ain Shams University (PHG-*p*-AR-309).

### 3.2. Apparatus

^1^H and ^13^C NMR experiments were carried out using a Bruker instrument (Billerica, MA, USA); 400 and 100 MHz, respectively. Results were expressed as δ ppm values and using TMS signal as an internal reference. UV absorbance was measured using a Shimadzu UV-1700 (Shimadzu, Kyoto 604-851, Japan). Mass spectra were recorded with a Finnigan MAT SSQ-7000 quadrupole mass spectrometer (ThermoFinnigan, Bremen, Germany). Data analysis was carried out with Xcalibur 2.0.7 (Thermo-Finnigan, Bremen, Germany).

### 3.3. Baicalein 5,6-Dimethyl Ether Extraction and Isolation

Air-dried leaves of *A. rugosa* (1.35 kg) were powdered and percolated in 70% methanol (8 L × 3), then filtered. The extract was evaporated to dryness under vacuum using a rotary evaporator and subsequently lyophilized to yield 100 g total methanol extract. The total extract was chromatographed on a Diaion HP–20 open column (120 ×10 cm), successively eluted with water followed by methanol: water (1:1), and finally methanol to yield 32 g water fraction; 10.8 g of 50% methanol fraction and 26.3 g of the methanol fraction. The methanol fraction (22 g) was chromatographed on a silica gel open column (120 × 5 cm). The column was eluted with dichloromethane (DCM), followed by a gradient mixture of DCM/MeOH up to 100% MeOH. A total of 120 fractions (200 mL each) were collected and analyzed using thin-layer chromatography (TLC) plates (silica gel F254) developed with n-hexane/EtOAc and DCM/MeOH as solvent systems; visualized under UV light (254 nm and 365 nm); sprayed with 10% H_2_SO_4_ and heated over a hot plate. Similar fractions were pooled together to yield 22 fractions (fractions AR-1 to AR-22). Compound **1** was crystallized from fraction AR-5 eluted with DCM and further purified on preparative TLC to yield compound **1** (74.22 mg).

### 3.4. Baicalein 5,6-Dimethyl Ether (1)

**Figure 6:**
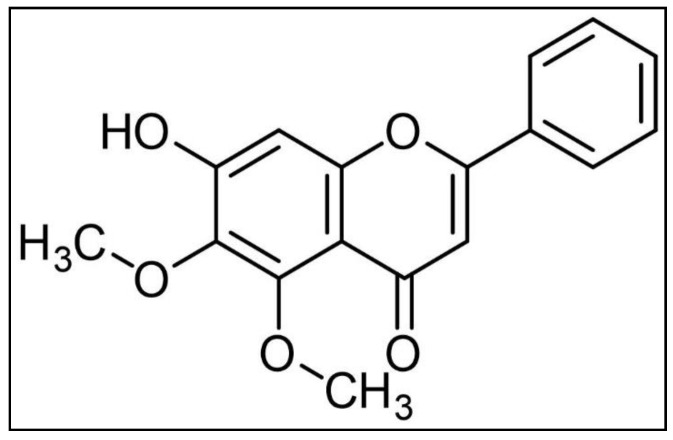
Chemical structure of compound 1, baicalein 5,6-dimethyl ether.

Yellow amorphous powder (74.22 mg), UV (MeOH) λ_max_ 267, 320; ^1^H NMR (500 MHz, Methanol-*d*_4_): *δ* 8.07 (m, 2H, H-2′,6′), 8.01 (m, 1H), 7.56 (m, 2H, H-3′,5′), 6.76 (s, 1H, H-3), 6.55 (s, 1H, H-8), 3.91 (s, 3H, 5-OCH_3_), 3.82 (s, 3H, 6-OCH_3_); ^13^C NMR (126 MHz; Methanol-*d*_4_): *δ* 180.51 (C-4), 162.65 (C-2), 157.63 (C-7), 154.05 (C-5), 153.80 (C-9), 140.28 (C-6), 131.81 (C-1′), 129.73 (C-3′, C-5′), 129.48 (C-2′, C-6′), 127.46 (C-4′), 106.58 (C-10), 105.61 (C-3), 95.16 (C-8), 60.96 (5-OCH_3_), 60.86 (6-OCH_3_). ESI-MS (*m/z*) 299.15 [M+ H]^+^, MS^2^ *m/z* 284 [M+ H-CH_3_]^+^, 281 [M+ H-H_2_O]^+^, 269 [M+ H-2CH_3_]^+^, 253 [M+ H-H_2_O-CO]^+^, 225, 197 [^1,3^A]^+^, as shown in Figure 6.

### 3.5. Experimental Animals and Treatment

**Figure 7:**
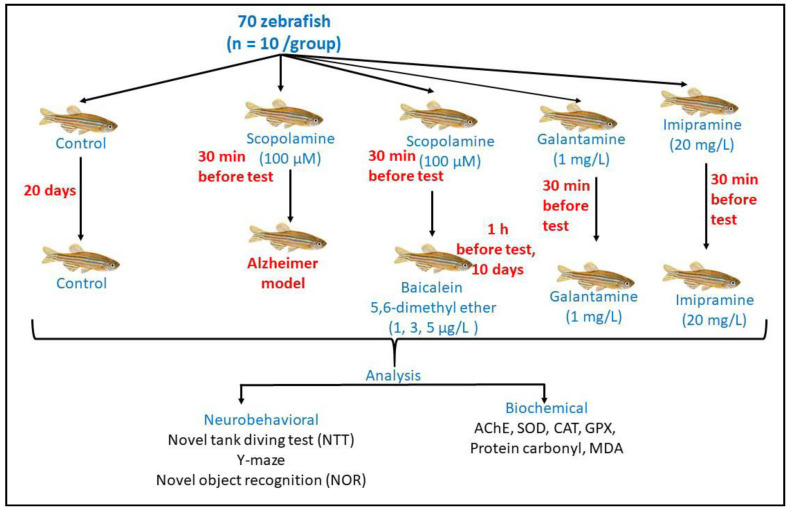
Schematic representation of the study experimental design, behavioral tests, and biochemical analyses.

Seventy adults of both sexes (3–4 months old), wild-type short-fin zebrafish (obtained from an authorized company: Pet Product S.R.L. Bucharest, Romania) were maintained in groups of 10 fish per 10 L freshwater home tank at 26 °C ± 2 with a controlled 14:10 h of the light/dark cycle. An adult zebrafish diet (Norwin Norvitall flake) was fed twice daily. A week before the experimental studies (Figure 7), fish were observed in quarantine and randomly divided into control, scopolamine (Sco, 100 µM), and three baicalein 5,6-dimethyl ether treatment groups (1, 3, and 5 µg/L).

Additionally, the galantamine (GAL, 1 mg/L) and imipramine (IMP, 20 mg/L) groups were used as positive controls. The baicalein 5,6-dimethyl ether [54] and Sco [55,56] doses were selected according to previous studies. Thirty minutes before performing the behavioral tests, all fish, except those in the control group, were individually immersed in Sco (100 µM) to induce the zebrafish model of amnesia, as previously described [54,57]. The baicalein treatment 5,6-dimethyl ether (1, 3, and 5 µg/L) was administered by immersion to Sco-treated zebrafish for 1 h before behavioral tests, once daily for 10 days. Also, GAL and IMP were administered by immersion 30 min before performing the behavioral tests in the Sco-treated fish. This study was conducted in strict accordance with the recommendations of the Directive 2010/63/EU of the European Parliament and of the Council of 22 September 2010 on the protection of animals. All the experiments in this study were conducted following the ethical approval of the Animal Ethics Committee of the Faculty of Biology, Alexandru Ioan Cuza University, Iasi, Romania (Project approval number: 02/30.06.2020).

### 3.6. Novel Tank-Diving Test (NTT)

To explore whether baicalein 5,6-dimethyl ether treatment affects locomotion or anxiety-like behavior, the animals were submitted to NTT, according to a previously described method by Levin et al. [58]. NTT is widely used to measure exploratory behavior and novelty stress habituation [59]. Zebrafish (*n* = 10 per group) were individually introduced in the apparatus filled with 1.5 L of home tank water and their behaviors were recorded for 6 min. Videos were analyzed using an automated video-tracking system (ANY-maze; Stoelting CO, Wood Dale, IL, USA) and a Logitech HD Webcam C922 Pro Stream camera (Logitech, Lausanne, Switzerland). The following endpoints were measured: time spent in the top (s), time spent in the top/bottom ratio, total distance travelled (m), and distance top/bottom ratio. In the NTT test, imipramine (IMP, 20 mg/L) was used as the reference drug.

### 3.7. Y-Maze Task

To explore the response to novelty as a result of baicalein 5,6-dimethyl ether treatment, a Y-maze was used following a method previously used by Boiangiu et al. [57]. Animals (*n* = 10 per group) were individually tested in the Y-maze with sides covered by black plastic self-adhesive film. A geometric cue (square, triangle, or circle) on the side of each arm allowed the fish to recognize each arm. Three liters of home tank water were used in the apparatus. The Y-maze arms were designated as follows: start arm (always open); novel arm (blocked during the first trial but opened during the second trial (test trial), and another arm (always open). The Y-maze center was not counted for the analyses. The Y-maze task consisted of two trials separated by an hour inter-trial interval (1 h ITI) to assess response to novelty [28]. During the first trial (training, 5 min), fish could explore only two arms (start and other), with the third arm (novel) closed. For the second trial (test trial after 1 h ITI), the fish were placed back in the same starting arm with free access to all three arms for 5 min. During 5 min (test trial), the following behavioral endpoints were analyzed: time in the novel arm (% of the total time), spontaneous alternation percentage, total distance travelled (m), and turn angle (°). Galantamine (GAL, 1 mg/L) was used as the reference drug in the Y-maze test. Training and test sessions were recorded using a Logitech HD Webcam C922 Pro Stream camera and further analyzed using an automated video-tracking system (ANY-maze; Stoelting CO, USA).

### 3.8. Novel Object Recognition Test (NOR)

NOR is a widely used behavioral assay in zebrafish to examine the memory efficiency [60]. The experimental apparatus consists of a 20 L glass tank (30 × 30 × 30 cm) filled with 6 cm of water. The NOR test consists of three phases. In the habituation phase, each animal explores the tank in the absence of the objects for 5 min twice a day (5 h interval between habituation sessions) over 3 consecutive days. In the training phase (on the 4th day), the animals were exposed to two identical hard plastic red cubes for 10 min. In the test phase (1 h after the training phase), a novel object (N, green cube) replaced one of the copies of the familiar objects (F, red cubes), and the exploration time of each object was evaluated for 10 min. The exploration area was established by increasing once the size of the object area; thus, we considered exploration when the fish were up to 2.5 cm far from each side of the object. The behavior was fully analyzed using the ANY-Maze^®^ software (Stoelting CO, Wood Dale, IL, USA), assessing the exploratory time (s) and the preference percentages. The preference percentages have been determined as (time of exploration of N/time of exploration of F + time of exploration of N × 100) [54].

### 3.9. Tissue Preparation

Following the behavioral tests, all fish were cryoanesthetized and euthanized by decapitation. The brain samples were dissected and gently homogenized (1:10) in ice-cold 0.1 M potassium phosphate buffer (pH 7.4), 1.15% KCl using a Potter Homogenizer (Heidolph Instruments, Schwabach, Germany) coupled with Cole-Parmer Servodyne Mixer (Cole-Parmer Instrument Co., Chicago, IL, USA). The supernatant was used for the assays of the acetylcholinesterase (AChE) [61], superoxide dismutase (SOD) [62], catalase (CAT) [63], and glutathione peroxidase (GPX) [64] specific activities, with protein carbonyl [65,66], and malondialdehyde (MDA) [67] levels. To determine the total protein, the method described by Smith et al. [68] was used.

### 3.10. Statistics

Data are expressed as the mean ± standard error of the mean (S.E.M) and analyzed using a one-way analysis of variance (ANOVA) followed by Tukey’s post hoc multiple comparison test. The level of significance was set at *p* < 0.05. Data were analyzed for normality using Shapiro-Wilk-Test. Pearson correlation analyses were used to assay the correlation between the behavioral scores, enzymatic activities, and lipid peroxidation. All values were calculated using GraphPad software (GraphPad Prism 7.0, La Jolla, CA, USA).

## 4. Conclusions

This study was conducted to evaluate the anxiolytic, antiamnesic, and antioxidant activities of baicalein 5,6-dimethyl ether isolated for the first time from *A. rugosa* in a Sco-induced amnesia zebrafish model. Our findings demonstrated that baicalein 5,6-dimethyl ether decreased anxiety and cognitive deficits, and increased exploratory behavior in specific tasks. Furthermore, in the Sco-treated zebrafish, treatment with baicalein 5,6-dimethyl ether-decreased AChE activity increased the antioxidant enzyme function and reduced the levels of oxidative markers (lipid and protein oxidation). The present results suggest that the underlying mechanism of memory improvement may include cholinergic system regulation and oxidative stress reduction. This evidence indicates that baicalein 5,6-dimethyl ether might be a viable therapeutic alternative for ameliorating dementia-associated conditions.

## Data Availability

The data presented in this study are available on request from the corresponding author.
